# Impact of operational temperature changes and freeze–thaw cycles on the hydraulic conductivity of borehole heat exchangers

**DOI:** 10.1186/s40517-021-00206-y

**Published:** 2021-10-28

**Authors:** Jan-Henrik Kupfernagel, Jan Christopher Hesse, Markus Schedel, Bastian Welsch, Hauke Anbergen, Lutz Müller, Ingo Sass

**Affiliations:** 1 Department of Environmental Engineering and Applied Computer Science, Geotechnical and Geothermal Engineering, Ostwestfalen-Lippe University of Applied Sciences, An der Wilhelmshöhe 44, 37671 Höxter, Germany; 2 Department of Materials and Earth Sciences, Geothermal Science and Technology, Technical University of Darmstadt, Schnittspahnstr. 9, 64287 Darmstadt, Germany; 3 Darmstadt Graduate School of Excellence Energy Science and Engineering, Technical University of Darmstadt, Otto-Berndt-Str. 3, 64287 Darmstadt, Germany; 4 Dipl.-Ing. Peter Neumann Baugrunduntersuchung GmbH & Co. KG, Marienthaler Str. 6, 24340 Eckernförde, Germany

**Keywords:** Borehole heat exchanger, Freeze–thaw cycles, Grouting material, Pilot-scale experiment, Hydraulic system conductivity, System integrity

## Abstract

A large share of the primary energy is consumed to provide space heating. Geothermal energy offers a regenerative alternative. For reasons of efficiency and environmental protection, it is important to ensure the system integrity of a borehole heat exchanger (BHE). Previous investigations have focused on the individual components of the BHE or on the grout and pipe systems’ integrity. This study focused on the analysis of the hydraulic system integrity of the complete subsoil–grout–pipe system as well as possible thermally induced changes. For this purpose, a pilot-scale experiment was built to test a 1-m section of a typical BHE under in situ pressure, hydraulic and temperature conditions. During the tests the hydraulic system permeability of the soil and the BHE was measured continuously and separately from each other. In addition, the temperature monitoring array was installed in a 50-cm cross-sectional area. Significant temperature-related fluctuations in the sealing performance could be observed. Hydraulic conductivity limits required by VDI 4640-2 (Thermal use of the underground—ground source heat pump systems, 2019) were exceeded without frost action. The succeeding application of freeze–thaw cycles further enhances the system permeability. The study shows that the thermally induced effects on the system integrity of the BHE are larger and more significant than the subsequent frost-induced effects. The hydrophobic character of the high-density polyethylene (PE-HD) pipes as well as its high coefficient of thermal expansion seem to be the main points of weakness in the system. Optimization research should focus on the interface connection between grout and pipe, whereby hydrophilic pipe materials such as stainless steel or aluminum should also be considered as well as manipulation of the pipe surface properties of PE-HD.

## Introduction

The reduction of greenhouse gas emissions is essential to counteract manmade climate change. Geothermal energy is a renewable energy source with a high potential for replacing fossil fuels. It can be used for heating and cooling purposes, which are both energy-intensive when using conventional energy sources (Rezaie and Rosen 2012). Thus, an increased use of geothermal energy can reduce CO_2_ emissions on a global scale (van der Zwaan and Dalla Longa 2019).

In the private house sector, the most common application to utilize geothermal energy are heat pump systems combined with borehole heat exchangers (BHE). Typically, drilling depths for such systems range from 70 to 200 m. These so-called shallow BHEs are in the focus of this study (Sass et al. 2016).

There are various ways to complete a BHE. However, the most common construction design, which is chosen as a reference type in this study, consists of two closed-loop polyethylene U-pipes inserted into a borehole and being grouted subsequently (Sass et al. 2016). The annulus is usually grouted from the bottom to the top with a cement-based suspension to seal-off the BHE. Both the vertical and horizontal hydraulic conductivity of a BHE system must be minimized to prevent vertical groundwater flow and thereby avoid aquifer interconnection and possible contaminant transport from the surface (Anbergen et al. 2014). The integrity of a BHE is of utmost importance for a sustainable system operation.

**Fig. 1:**
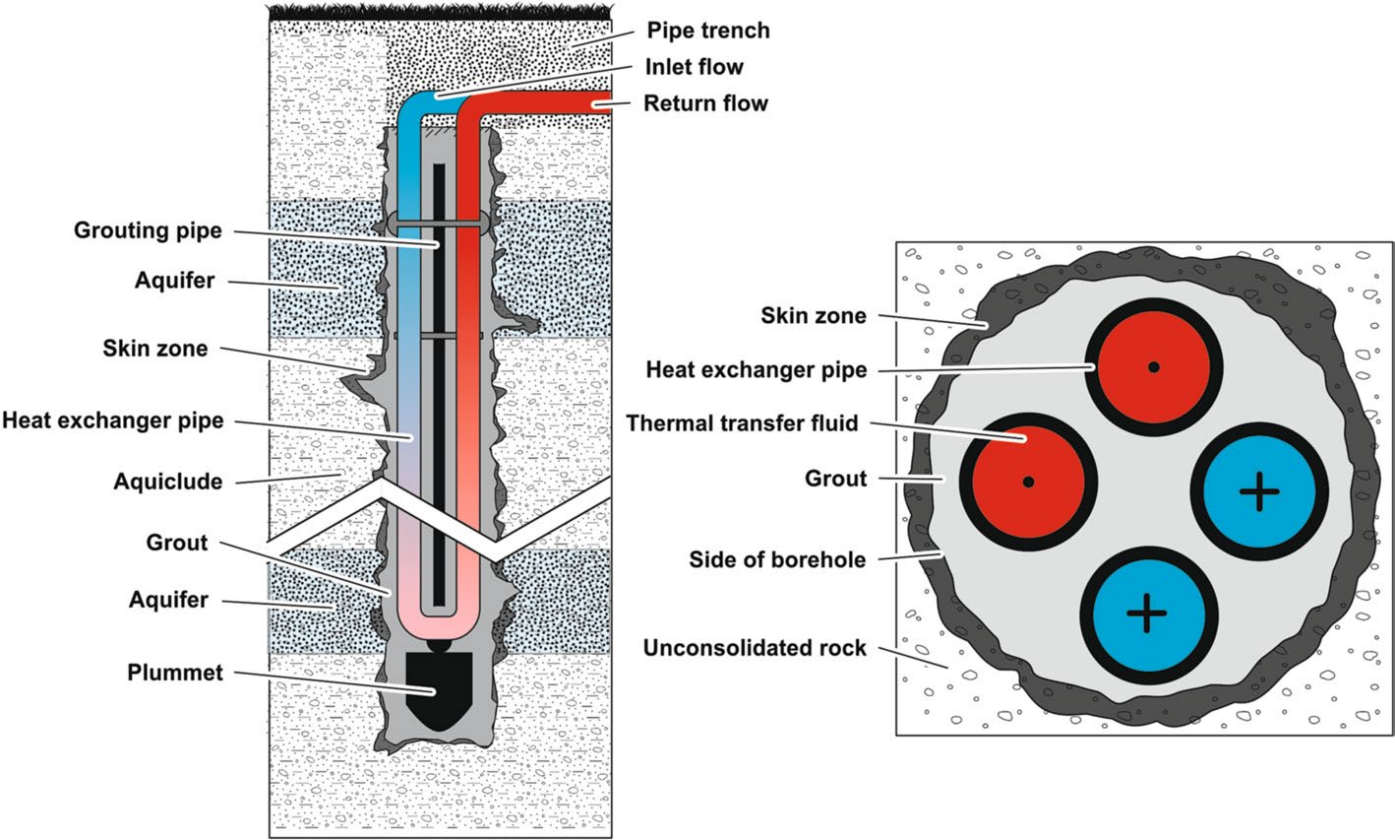
Left: schematic sketch of a typical double U-pipe BHE system (only one of the two congruent U-pipes is depicted). The borehole penetrates two aquifers and is filled with grout. Right: schematic view in horizontal section (modified after Sass et al. 2016)

Due to the drilling operation, the vicinity of the borehole is mechanically influenced forming a so-called skin zone with possibly altered material properties (Novakowski 1989). Accordingly, the BHE system can be subdivided from outside to inside into the undisturbed underground formation, the skin zone and the grouting material surrounding the heat exchanger pipes (Fig. 1). Consequently, the grout has to seal both, the borehole itself as well as the skin zone.

Since the natural temperatures of the shallow subsurface are in most cases too low for direct space heating, a heat pump is needed to provide the required supply temperatures of the space heating. During peak loads, the heat extraction from the BHE can exceed the heat flux from the surrounding rock formation leading to a temperature decrease within the BHE and its vicinity even below the freezing point (Sass et al. 2016). To prevent freezing of the heat carrier fluid, which is pumped through the pipes, the fluid is usually composed of a mixture of water and anti-freeze admixtures such as ethylene–glycol.

The thermal efficiency of a BHE system strongly depends on its thermal connection to the surrounding natural subsurface. In other words, the thermal transfer resistances between the heat carrier fluid inside the pipe and the natural subsurface must be as low as possible. This can be achieved, on the one hand, by high thermal conductivities of the pipe and grout materials and, on the other hand, by an intact crack- and gap-free pipe–grout–underground body.

Due to the higher investment costs compared to similar heating systems, such as air source heat pumps, BHE systems have to achieve long lifetimes with a fault-free operation to be economically competitive (Blum et al. 2011). This presupposes that both the sealing function as well as the thermal properties must remain constant over its operational lifetime. It implies that a BHE should be designed to withstand mechanical stress and environmental impacts under all occurring working conditions. However, in particular the exposure of the system to cyclic freeze–thaw loads is seen as a leading cause for a potential impairment of the system’s integrity (Anbergen et al. 2014).

The general occurrence, the magnitude and duration of freezing events in a BHE system depend on two main factors: the sizing of the BHE and the magnitude of the thermal load. Consequently, an undersizing of the system as well as an unexpectedly high heat demand facilitate freezing in the vicinity of the BHE (Sass et al. 2016).

The effects of freeze–thaw cycles (FTC) on a complete BHE system including the surrounding soil have not been investigated experimentally to date. This study presents a new experiment that applies FTC on an in situ BHE specimen at pilot scale (realistic circumferential geometry, 1-m vertical section) under realistic soil mechanical and hydrogeological conditions. The sampling and monitoring design of this newly developed triaxial vessel is able to research integrity changes for individual construction components as well as the hydraulic performance of the whole system. The inclusion of the surrounding soil as well as the possibility to determine individual flows within the sample are a novelty to previous investigations of a BHE system.

## Freeze–thaw loads on BHE systems

Investigations of BHE system integrity and freeze–thaw cycles present a complex issue. The development of a pilot-scale test on this topic must take into account the implementation of theoretical approaches, guideline requirements, and existing small-scale laboratory tests. These fundamentals are reviewed in more detail in the following sections.

**Fig. 2:**
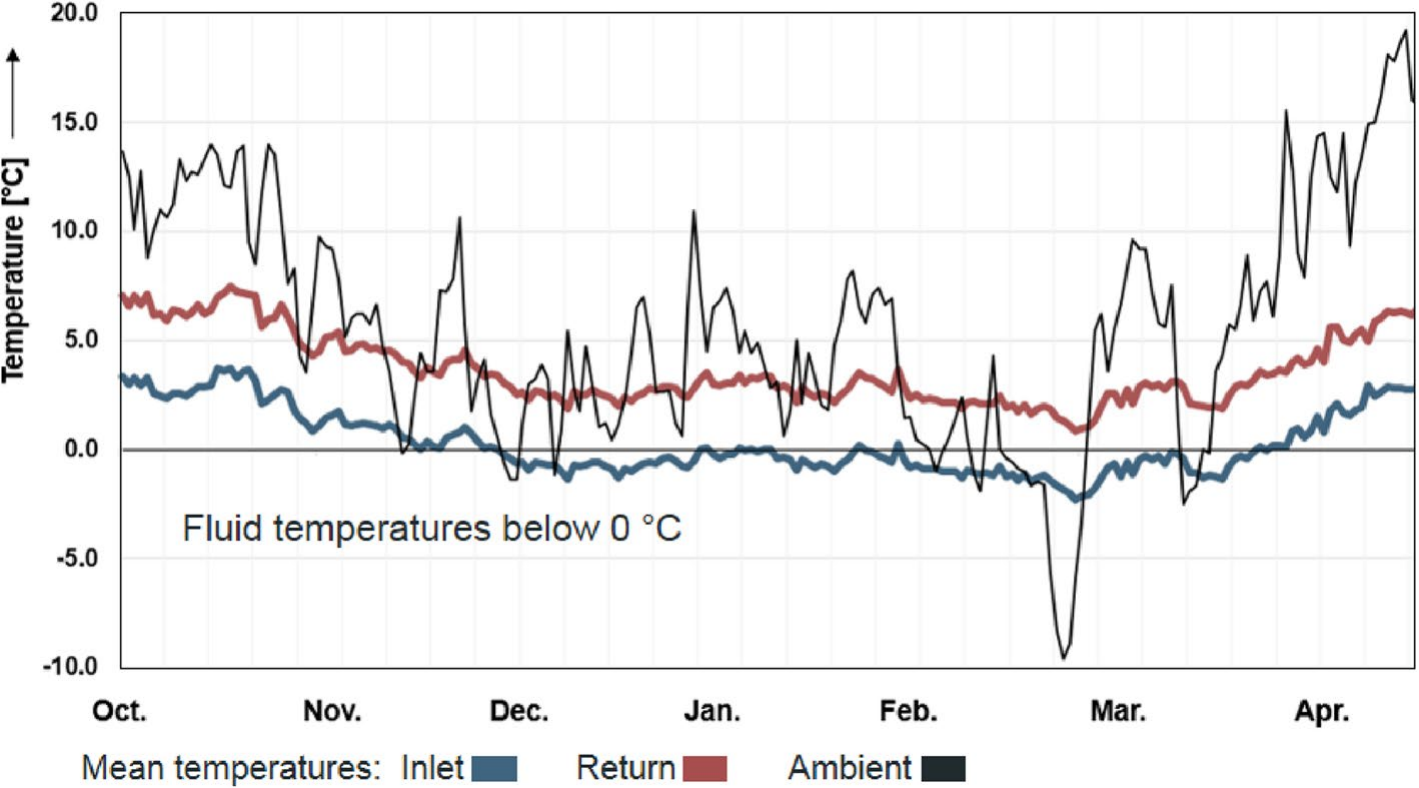
Daily mean temperatures of outlet and inlet flow of an exemplary BHE near Stuttgart (Germany) in winter 2018 and the related ambient temperature

According to the German guideline VDI 4640-2 (2019), BHE systems must be designed depending on the building heating curve and an intended operating period (minimum 50 years). In this period, the monthly mean temperature of the BHE inlet flow should not fall below 0 °C. Peak load temperatures should not drop below − 5 °C. However, the operational parameters are barely monitored in practice. Moreover, during the planned operating time heat loads of buildings might vary due to changes in user behavior or an enlargement of the living space. Thus, an enervation of BHE systems can never be completely avoided for their entire lifetime. As exemplified in Fig. 2, this can lead to temperatures of the inlet flow below 0 °C for several months—and this despite comparatively warm ambient temperatures during that period. Only after the thermal load is significantly reduced, the system recovers and leaves freezing–thawing conditions.

The formation of ice in porous media such as grouting material or soil is a complex process resulting from coupled heat and mass transfer of the porous media’s solid and fluid components (McKenzie et al. 2007). During heat extraction, the temperature in the vicinity of the heat exchanger pipes of a BHE is progressively reduced. Once the temperature in the grouting material drops below the freezing point of the pore water, the pore water freezes and as a consequence, its volume increases. As soon as ice nucleation occurs in the capillaries, the frost suction causes additional water flow towards the nucleus which leads to the formation of ice lenses. This process increases with increasing distance from the frost source (Konrad and Morgenstern 1980). Below the phreatic surface, the grout in a BHE system is saturated and the mechanical pressure on the grout structure increases during the freezing process. As soon as the tensile strength of the material is exceeded, cracks will form. Such fissures significantly increase the grout’s hydraulic conductivity (Anbergen et al. 2014). The same effect can occur in the subsoil (Chamberlain and Gow 1979; Dalla Santa et al. 2019a; Othman and Benson 1993; Sterpi 2015).

Consequently, cracking induced by FTC can cause serious damage to a BHE system up to a complete failure of system integrity (Anbergen et al. 2014).

There are several approaches to overcome this problem. German authorities tend to avoid inlet temperatures below 0 °C, which has negative effects on the efficiency and flexibility of system operation. Another option is to restrict the installation of BHE to freezing–thawing resistive rocks such as granites. In countries like Sweden, Finland and Norway with vast granitic outcrops borehole heat exchangers are usually not sealed off. Examples of different country-specific requirements for grouting material can be found in Table 1.

**Table 1:** Requirements to grouting materials and BHE sealing modified after IEA ECES (2020)

Belgium	The permeability needs to be less than 10^–8^ m s^−1^. Mostly cement–bentonite grouts and clay pellets are used
Denmark	The only requirements are related to the sealing properties. So far only bentonite-based grouts have been used in DK. The legislation demands “impermeable materials”. This means that materials other than bentonite, with the same permeability properties, may be allowed
Finland	Not specified
Germany	In almost all cases a cementitious grouting slurry is used. Rarely are swelling clay pellets used for grouting in case of fissures in combination with groundwater. Thermal enhanced clay pellets and a special pumping device have been developed. The permeability of the backfill needs to be ≤ 10^–10^ m s^−1^ according to the VDI 4640 guideline. However, this value is under discussion in Germany
Japan	Not specified
Netherlands	The permeability needs to be less than 10^–9^ m s^−1^. Clay from the drilling itself cannot be used
Sweden	Alternative sealing by different forms of sealing plugs that are attached to the collector. These are used to avoid salt or brackish water to enter higher levels in the boreholes, but also to seal-off potential leakage between aquifers
Turkey	Not specified

Moreover, it would also be an option to limit the installation of BHEs to aquifers that do not suffer any damage or cause any damage in the event of hydraulic short circuits. However, all limitations to certain geologic units would limit the regional distribution and thus the overall potential of BHE systems severely. A detailed investigation of the governing damaging processes combined with the development of technical solutions such as modified heat exchangers or grouting materials seems to be a more reasonable approach.

### Investigation of BHE system integrity

There have been a number of investigations in system integrity of BHE in the past. Initial research on the influence of the interaction of grouting materials and pipes on the overall hydraulic conductivity was made on groundwater well construction materials (Edil et al. 1992). An important finding already was that the hydraulic conductivity of the complete system is higher than of the simple grouting material property (Allan and Philippacopoulos 1998). Due to the similarities in the technical construction of both applications, the experiments were extended to pipe and grouting materials used in geothermal systems. It was found that temperature-induced expansion and contraction of plastic heat exchanger pipes, in combination with cement–sand grouts, can have an influence on the hydraulic system conductivity as well as the bonding strength (Allan 2000). Furthermore, modeling results of thermal influences on the gap between pipe and grout that made indications of changes in the apparent thermal conductivity when the pipes are contracted (Philippacopoulos and Berndt 2001).

Initial research on the impact of FTC on BHE systems first dealt with the behavior of the grouting material performance under cyclic frost loads in accordance to standards specified for concrete or natural rocks such as DIN 52104-2 (1982) and DIN EN 1367-1 (2007). It became evident that cyclical frost loads have an undesired impact on the material strength of BHE grouting materials (Müller 2009). Further studies focused on the influence of FTC on the hydraulic conductivity and the sealing function of the grouting material, respectively, with a special focus on system analysis. Anbergen et al. (2014) developed a laboratory testing device to measure the vertical hydraulic conductivity of a system specimen consisting of a central heat exchanger pipe and surrounding grout. The experimental procedure was later integrated into the German guideline VDI 4640-2 (2019). Key findings of Anbergen et al. (2014) were that the system approach is essential for transferable results and the direction of frost propagation, similar to the real scale, is very important for the grade and geometry of possible freezing cracks. For the laboratory experiment, it was also shown that if frost damage in a grouting material occurs, it will happen within the first five FTC, while additional FTC do not add measurable damage to the system. These observations were generally confirmed by further experimental work, which carried out system tests with BHE configurations consisting of heat exchanger pipes and grout on real scale (Kirschbaum et al. 2018). Furthermore, Anbergen et al. (2015a) concluded that it is crucial to consider the latent heat in transient simulations of the FTC in water-saturated porous media.

The necessity of evaluating the whole BHE system, consisting of heat exchanger pipe, grout and surrounding subsoil, was only recently identified. Results of numerical simulations demonstrated that the frost front originating from the heat exchanger pipes propagates also into the surrounding soil and/or rock (Anbergen et al. 2015b; Dalla Santa et al. 2019b). Preliminary modeling testified that also in a single BHE system, freezing and thawing in the undisturbed host rock has to be considered. Consequently, the role of FTC-stress in this region must also be included in all integrity considerations. However, the impact of FTC on the hydraulic system conductivity cannot be assessed by numerical simulations without having experimental data to validate it. This necessitated the development of a pilot-scale experiment capable of investigating the relevant processes.

## Methods

In order to achieve the highest possible validation of the experiment, the following requirements for the design of the experiment were formulated in advance:

- Conformity of the specifications to ASTM D-5084-16a (2016).
- Consideration of asymmetrical thermal load due to different inlet and outlet temperatures in the BHE.
- Adjustable temperature on the outside of the sample.
- Monitoring of the temperatures in the horizontal specimen cross section.
- Separated monitoring (split-flow monitoring) of the main construction components of the BHE and the surrounding soil.

These specifications led to the development of the experimental setup described below.

### Pilot-scale experimental setup

**Fig. 3:**
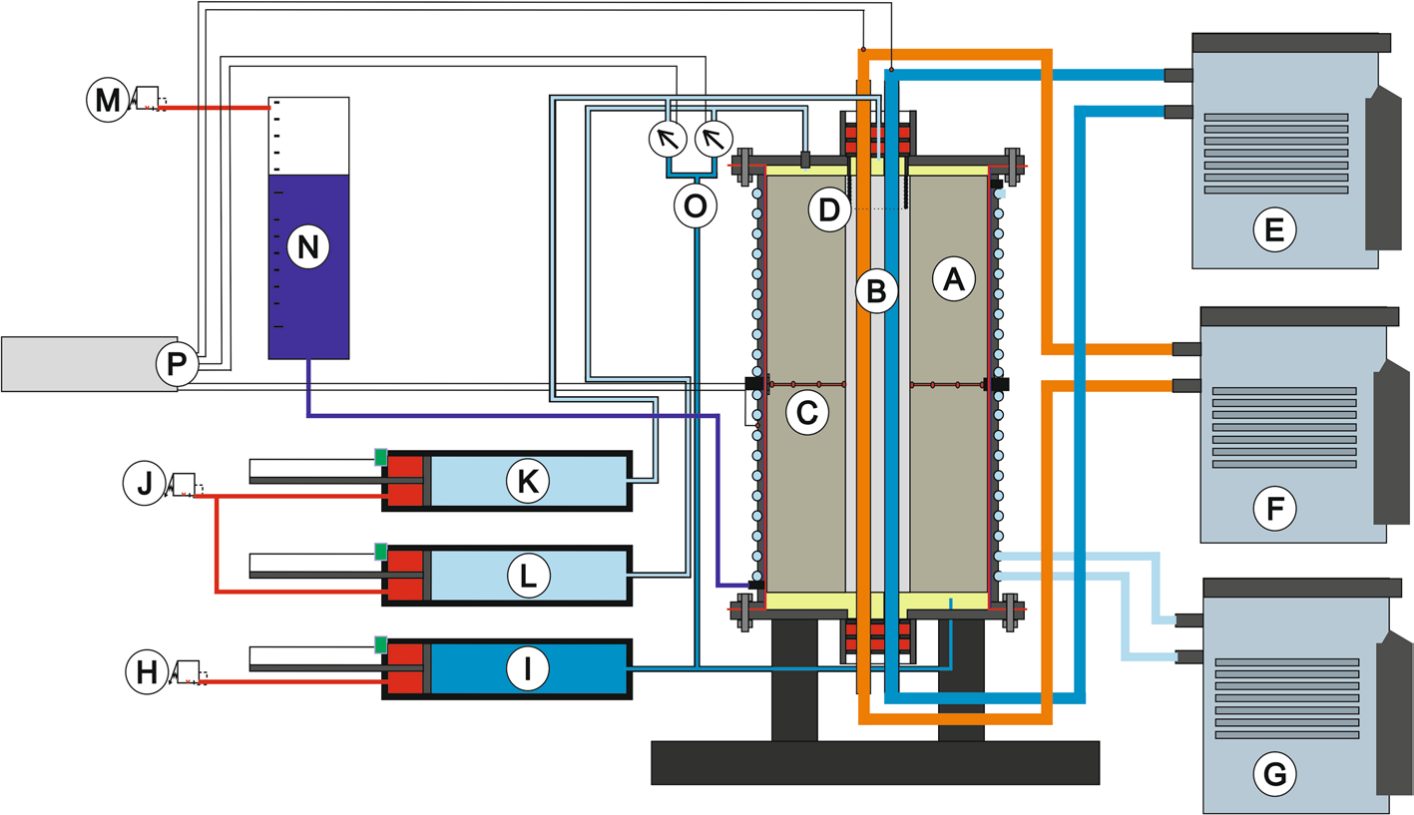
Schematic sketch of the pilot-scale experiment. Test vessel with internal latex sleeve, soil filling (A) and grouted double U-pipe BHE (B). Temperature sensors (C), flow path separation (D), inlet- and return flow temperature control of the BHE carrier fluid (E, F), temperature control of tank shell (G). Pneumatic pressure control (H inflow, J outflow and M cell pressure), hydraulic conductivity measurement via position tracking of hydraulic cylinders between flow approaching the sample from below (I) and separated return flow (L, K). Cell pressure and measurement of displacement (N), differential pressure measurement (O) and data logger (P). Not shown: external thermal insulation

The central device of the pilot-scale experiment (Fig. 3) is a sealed cylindrical steel tank that can be operated with an internal working pressure of up to 1000 kPa. Preliminary numerical simulations formed the basis for dimensioning the tank with a height of 1000 mm and a diameter of 500 mm. Practical issues such as the time required to saturate the sample and limiting influences due to undefined temperature boundary conditions were the main deciding factors for this dimension. The operational modus of the tank system corresponds to a triaxial device according to ASTM D-5084-16a (2016) and DIN EN ISO 17892-11 (2019).

A latex sleeve between the tank wall and the specimen body allows to apply radial earth pressure. Furthermore, the sleeve seals off the soil sample to avoid interflow between specimen and vessel wall. At the outside of the steel wall, a spiral copper pipe heat exchanger is installed and connected to a thermostat. The tempering of the steel wall as outer boundary condition is handled with four separated counterflow circuits. The inlet and return flow of the BHE are connected to a high-performance thermostat each, so that the asymmetric heat extraction can be carried out under field-like conditions. The thermostat´s regulation range is from − 20 to 40 °C. Experimental operation took place between − 5 and 12 °C. This range can be regarded as realistic for typical BHE operation on site.

### Assembly of the pilot BHE

**Fig. 4:**
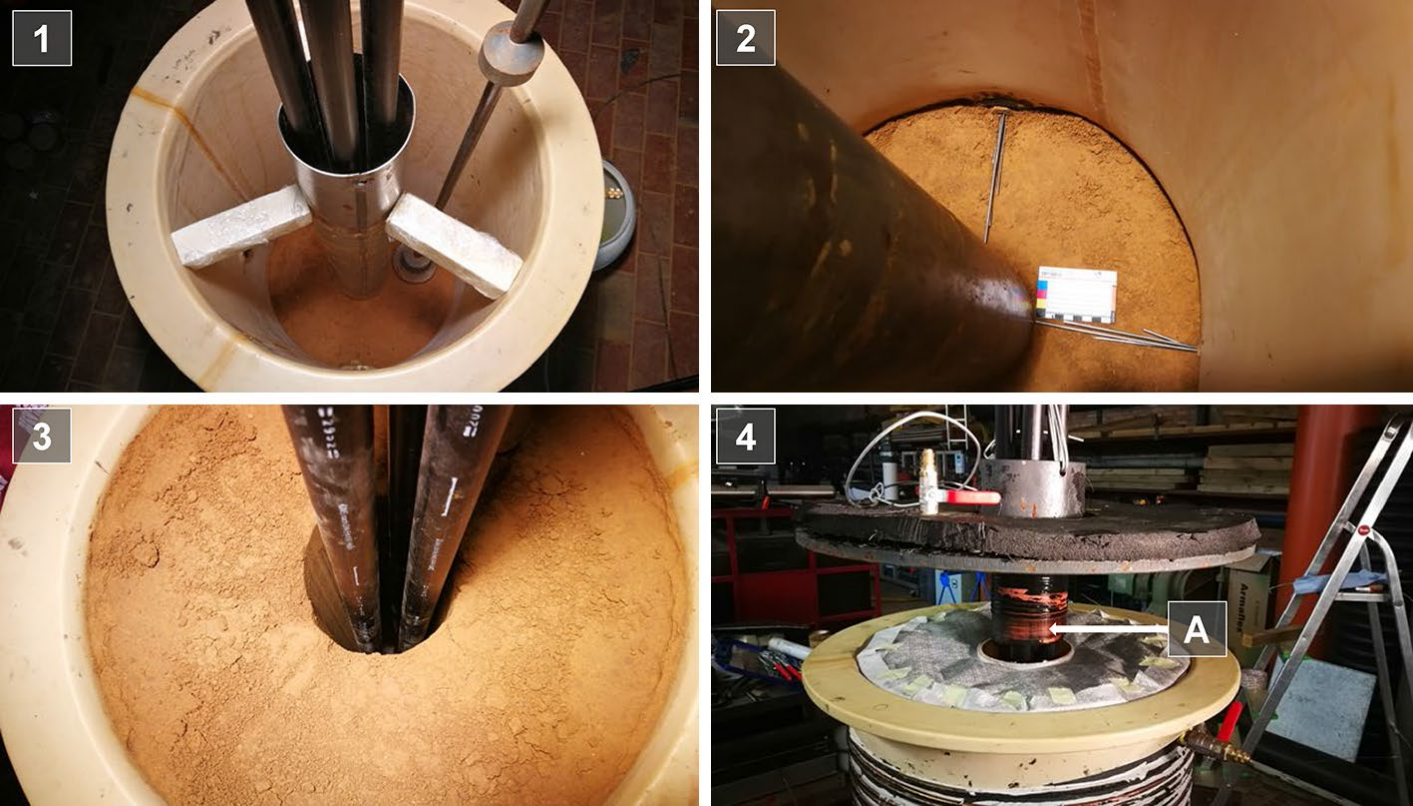
Completion of the experimental BHE. (1) Steel vessel covered with latex sleeve and centered tube; (2) vessel inside view with latex and center tube in place, installation of the temperature sensors; (3) after withdrawal of the center tube the compacted soil sample stays in place; (4) closure of the vessel after installation of the top drainage layer. The detachment of the paths to monitor and operate the flow is indicated with arrow A

For the assembly of the experiment, a steel pipe (*Ø*_out_ 150 mm) was centered as a standpipe in the vessel first. The previously selected soil was filled in the annulus around the standpipe and compacted at proctor water content to achieve a reasonable consolidation state. As especially in aquitard horizons, vertical hydraulic integrity is essential for the permissible operation of BHE, a loamy soil consisting of silt, clay and fine sand (*U*, *t*′, fs′) with low hydraulic conductivity was selected to represent these conditions. Four BHE pipes of PE-HD (*Ø*_out_ 32 mm) were placed inside the standpipe. This represents the most common double U-pipe BHE configuration in Germany (Sass et al. 2016). In the next step, the standpipe was withdrawn and immediately the free space between the PE pipes and soil was grouted with a cement-based suspension. This arrangement well approximates a real double U-pipe BHE geometry with regard to the horizontal cross section. The experimental BHE is based on a bottom drainage layer (basalt cuttings of 1–3 mm in diameter) and covered by a top drainage layer. A hydrophilic tissue filter sheet separates the drainage from the soil. Moreover, the grout suspension hardened for 56 days in the closed and sealed tank at a temperature of 12 °C (undisturbed soil reference temperature). Illustrations of the construction procedure are given in Fig. 4. The properties of the used soil, grouting and pipe materials are summarized in Table 2.

**Table 2:** Properties of the soil, grouting and pipe material used in the experiment

Soil				
Dry density	DIN EN 13286-1 (2003)	*ρ*_d_	1.71	g cm^−3^
Grain density	DIN EN ISO 17892-3 (2015b)	*ρ*_s_	2.67	g cm^−3^
Porosity	DIN EN ISO 17892-2 (2015a)	*Ф*	~ 36	%
Hydraulic conductivity	DIN EN ISO 17892-11 (2019)	*K*	1 × 10^–8^	m s^−1^
Thermal conductivity (Sat.)	Drefke et al. (2017)	*λ*	2.3	W m^−1^ K^−1^
Liquid limit	DIN EN ISO 17892-12 (2020)	*W*_L_	23.5	%
Plastic limit	DIN EN ISO 17892-12 (2020)	*W*_P_	21.3	%
Plasticity	DIN EN ISO 17892-12 (2020)	*I*_P_	2.2	%
Grout				
Suspension density	VDI 4640-2 (2019)	*ρ*_s_	1.47	g cm^−3^
Marsh funnel time (4.76 mm)	DIN EN ISO 10414-1 (2009)	*t*_f_	51	s
Water bleeding (24 h)	VDI 4640-2 (2019)		3	%
Uniaxial comp. strength (56 d)	DIN 18141-1 (2014)	*C*_u_	5.47	N mm^−2^
Hydraulic conductivity	DIN EN ISO 17892-11 (2019)	*K*	< 1 × 10^–11^	m s^−1^
Hydraulic system conductivity (FTC_0_)	VDI 4640-2 (2019)	*K*	7.0 × 10^–10^	m s^−1^
Hydraulic system conductivity (FTC_6_)	VDI 4640-2 (2019)	*K*	2.8 × 10^–9^	m s^−1^
Thermal conductivity	Manufacturer data	*λ*	> 2	W m^−1^ k^−1^
Pipe				
Pipe material	Manufacturer data	Polyethylene PE 100-RC		
Outer/inner diameter	Manufacturer data	*d*_o_/*d*_i_	32/26	mm
Wall thickness	Manufacturer data	*s*	3.0	mm
Surface roughness	Manufacturer data	*R*_t_	1.86	µm
Thermal conductivity	Manufacturer data	*λ*	0.40	W m^−1^ K^−1^
Coefficient of thermal expansion	Manufacturer data	*α*	180 × 10^–6^	K^−1^

### Hydrogeological conditions in the experiment

**Fig. 5:**
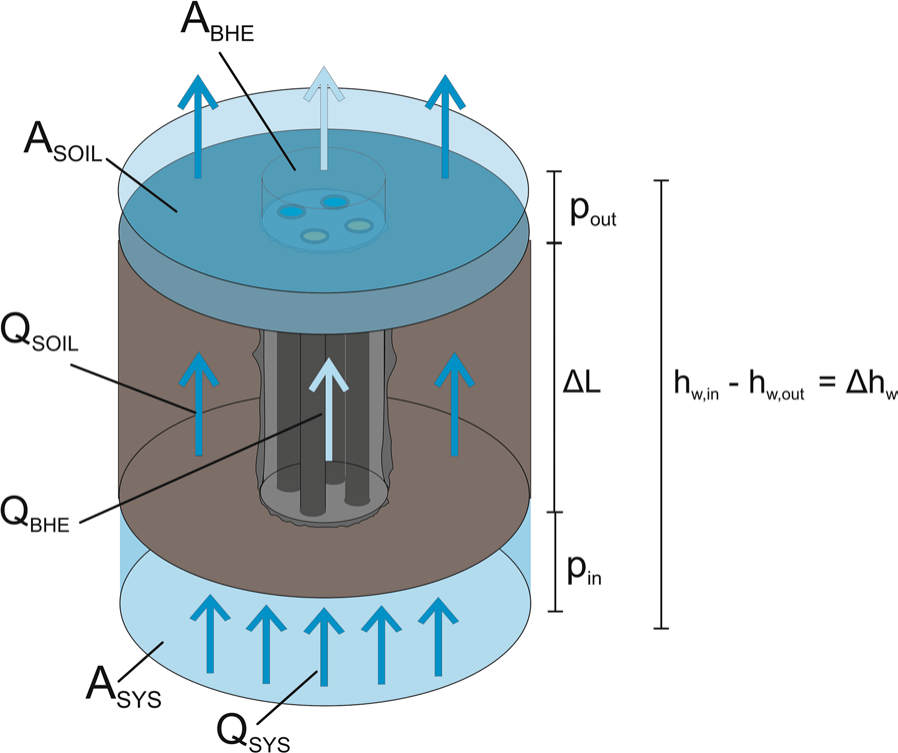
Sketch of the separated flows and cross-sectional areas. With Δ*L* as sample length, arrows are indicating the flow direction, Δ*h* as upwards hydraulic gradient (*Q*_SKIN_, *Q*_GROUT_ and *Q*_GAP_ are not displayed)

The flow direction during the hydraulic testing is from the bottom to the top, and water flow through the full cross-sectional area is ensured. At the top of the vessel the water is discharged by two independent pipes. Both extraction points were operated at the same pressure. Thus, the water volume flow through the soil (*Q*_soil_) and through the grout (*Q*_BHE_) can be determined independently (Fig. 5).

To separate the part-flows effectively, a roughened PVC pipe (Figs. 3D, 4A) was placed and sealed off in the center of the tank lid. This pipe acts as a flow splitter. It was embedded since the grout was still in the liquid state and the vessel was closed afterwards.

Three cylinders using compressed air were used to apply the hydraulic gradient to the sample. They are equipped with a displacement sensor on the stamp so that the piston position can be recorded and logged. In this study, the hydraulic gradient was build up by using the back-pressure method (ASTM D-5084-16a 2016). The specimen was wrapped with a latex sleeve to avoid circumferential flow. For this purpose, the latex sleeve is pressed onto the soil sample by a pneumatically regulated radial cell pressure representing the earth pressure horizontal tension (*σ*_2_ = *σ*_3_).

The hydraulic conductivity *K* [m s^−1^] is derived from Darcy’s Law as shown in Eq. 1, where v is the area related flow rate in m s^−1^, *i* is the hydraulic gradient over the sample, *A* is the cross-sectional area in m^2^, *Q* the mass flow in m^3^ s^−1^, Δ*h*_w_ the height difference of the water head in m_wh_ and *L* the length of the specimen in m:

1 $$K = \frac{v}{i} = \frac{Q \cdot L}{{\Delta h_{{\text{w}}} \cdot A}}$$

In order to calculate the different hydraulic conductivities for the soil and heat exchanger section, respectively, the sample surface *A* as well as the occurring mass flow *Q* must be specified according to their respective system areas (Fig. 5), as shown in Eqs. 2 and 3.

2 $$A_{{{\text{sys}}}} = A_{{{\text{soil}}}} + A_{{{\text{BHE}}}} = A_{{{\text{soil}}}} + A_{{{\text{skin}}}} + A_{{{\text{grout}}}} + A_{{{\text{GAP}}}} - A_{{{\text{pipes}}}} $$

3 $$Q_{{{\text{sys}}}} = Q_{{{\text{soil}}}} + Q_{{{\text{BHE}}}} = Q_{{{\text{soil}}}} + Q_{{{\text{skin}}}} + Q_{{{\text{grout}}}} + Q_{{{\text{GAP}}}} $$

An important functionality of the pilot-scale experiment is that the individual shares of *Q*_*i*_, dependent on each compartment of *A*_*i*_, can be monitored independently from each other (Eqs. 2 and 3). This allows to obtain individual proportionate hydraulic conductivities at any point and time of an experiment. The analysis of these isolated *K*-values serves to evaluate the integrity of each construction compartment. The conductivity of the overall system (*K*_SYS_) is calculated with Eq. 4. The factor *α*_T_ corrects the temperature-related variation of water viscosity according to ASTM D-5084-16a (2016):

4 $$K_{{{\text{sys}}}} = \frac{{L \cdot \left( {\Delta V_{{{\text{soil}}}} + \Delta V_{{{\text{BHE}}}} } \right) \cdot \alpha_{{\text{T}}} }}{{\Delta h_{{\text{w}}} \cdot \Delta t \cdot \left( {A_{{{\text{BHE}}}} + A_{{{\text{soil}}}} } \right)}}$$

with *K*_sys_ is the hydraulic conductivity of the system (m s^−1^); *L* is the sample length (m); ∆*V*_soil_ is the measured water flow volume in the soil fraction (m^3^); ∆*V*_BHE_ is the measured water flow volume in the BHE fraction (m^3^); *α*_T_ is the temperature correction factor for water viscosity (–); ∆*h*_w_ is the height difference of the water column between outlet and inlet (m_wh_); ∆*t* is the time (s); *A*_soil_ is the sample area of the soil fraction (m^2^); *A*_BHE_ is the sample area of the BHE fraction (m^2^).

The accuracy of the hydraulic conductivities determined with the tank experiment was calculated according to the Gaussian error propagation based on the individual uncertainties of the respective input variables. Table 3 summarizes the uncertainties of the input variables.

**Table 3:** Uncertainties of the input variables for the calculation of the hydraulic conductivities in the tank experiment

Sample length	0.001	m
Sample radius	0.001	m
Water flow volume	1.0 × 10^–6^	m^3^
Temperature of fluid	0.1	K
Height difference of the water column between outlet and inlet	0.1	m_wh_
Time	0.1	s

**Fig. 6:**
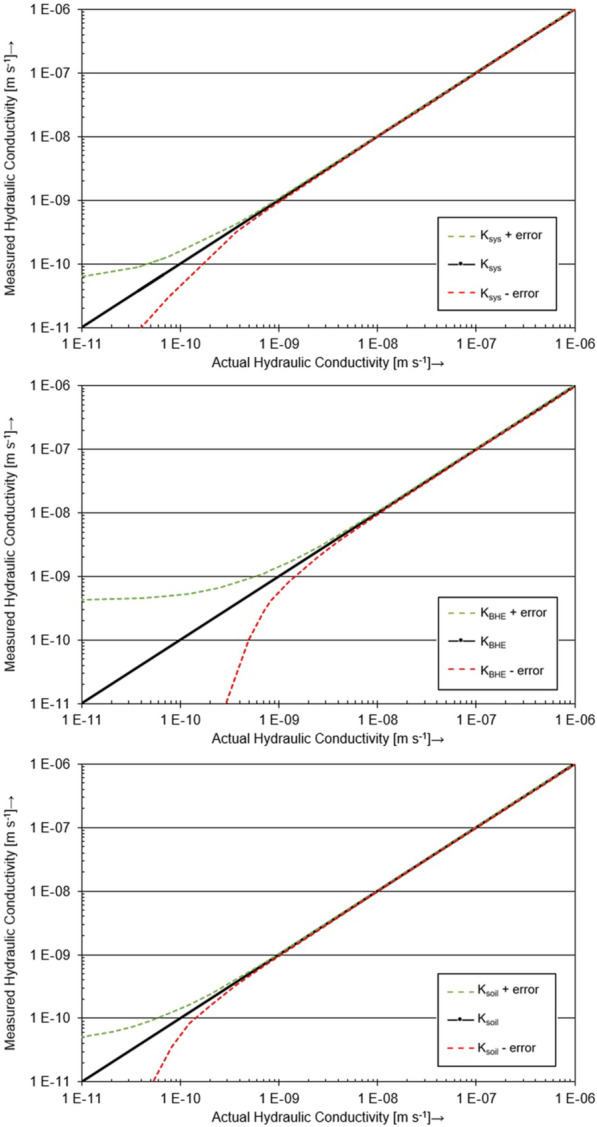
Overall uncertainties of the different hydraulic conductivity measurements with the tank experiment

In particular, the uncertainty of the water volume measurement has a major impact on the accuracy of the hydraulic conductivity results. Thus, maximum deviations apply especially to measurements of low hydraulic conductivities due to the lower volumetric water flow. Figure 6 shows the overall uncertainties of the various measurements of hydraulic conductivity obtained by the tank experiment. Due to the proportions of the area fractions in the sample cross section, measurements of the hydraulic permeability of the BHE column (*K*_BHE_) have the most significant uncertainties in this experiment. However, this has only a minor impact on the overall system hydraulic conductivity *K*_sys_.

### Thermal monitoring

**Fig. 7:**
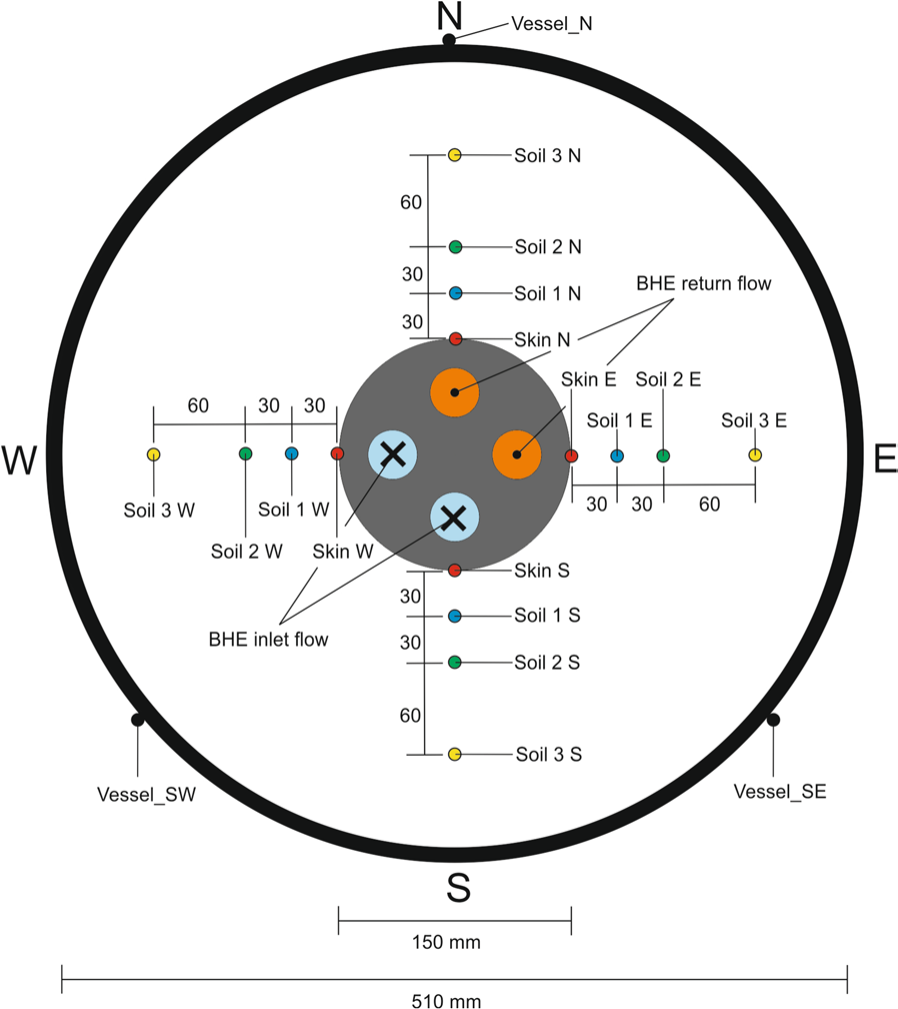
Schematic cross section of the pilot-scale experiment with sensor positions. Skin (red) is positioned in the skin zone, Soil (blue) BHE + 30 mm, Soil 2 (green) BHE + 60 mm, Soil 3 (yellow) BHE + 120 mm. BHE_in_ (orange) and BHE_out_ (blue) sits at the inlet of the pipe. Vessel_N, Vessel_SW and Vessel_SE (black) are located at the outside of the tank (all sensor positions are projected into the shown cross section plotted herein)

Type T thermocouples (calibrated to accuracy of ± 0.1 K) were installed (Fig. 7) in the soil in the middle of the sample height (500 mm) to investigate the radial temperature distribution in the vicinity of the BHE during the experiment. The sensors had to be installed orthogonal to the direction of flow to avoid a disturbance of the soil hydraulic conductivity measurements. For this purpose, they had to be inserted passing the tank and the latex sleeve, horizontally. 16 sensors were installed and distributed in the four cardinal directions around the BHE body. The deviations of these measurements in all spatial directions were below 5 mm. More sensors were installed in the inlet and return lines of the BHE pipes as well as at the external vessel wall.

### Test routines

Before the actual measurements started, a stationary hydraulic regime of sample hardening and water saturation under constant physical conditions had been established for at least 86 days. The first 56 days of that time span, the grout was allowed to cure and simultaneously the specimen was watered gravitationally using demineralized and de-aired water according to ASTM D-5084-16a (2016) and DIN EN ISO 17892-11 (2019). Starting on day 56, a hydraulic gradient (*p*_in_ 200 kPa, *p*_out_ 140 kPa, cell pressure 250 kPa) was applied and a water flow was initiated to saturate the specimen. Within the next 30 days, stationary flow conditions were established and full saturation conditions achieved. From day 87 on, the actual investigations presented in this study started.

Prior to the start of the first FTC measurements with a freezing of the specimen, the effect of temperature changes on the hydraulic integrity of the system above 0 °C were investigated. Thus, effects could be detected and quantified under ice-free operation. Differences of the coefficient of thermal expansion of the BHE pipe and grouting material in combination with temperature changes caused mechanical movements at the grout/pipe interface triggering proposed effects onto the hydraulic system conductivity (Allan 2000). Thus, both the inlet and return flow of the BHE were stepwise reduced from 12 to 3 °C (12, 10, 8, 5, 3 °C) and changes in hydraulic conditions were monitored for 10 h at each step. Afterwards the procedure was repeated in reversed order with a stepwise temperature increase back to 12 °C. The complete procedure was repeated again after finalizing the FTC testing of the experiment.

To investigate the influence of FTC on the hydraulic system integrity of BHE, a sequence of FTC then was applied in the pilot-scale experiment. Such a sequence is defined by a row of six single FTC in accordance to VDI 4640-2 (2019). The respective scheme for temperature control is summarized in Table 4. The inlet and return temperatures of − 3 and − 2 °C, respectively, are based on previous modeling results for the section after 10 years of operation with the highest horizontal spreading of the ice formation into the surrounding unconsolidated rock. In designing the experiment, attention was paid to ensure that the thawing of the specimen starts at the outside as in BHE systems in natural underground conditions. The parameters necessary to calculate the hydraulic conductivity were measured minute-wise continuously and served to calculate a representative *K*_*i*_ value for each FTC.

**Table 4:** Thermal routine scheme of one freeze–thaw cycle (FTC) out of a FTC sequence in the pilot experiment with constant hydraulic monitoring

Pre-conditioning	Freezing state	Thawing state	Cycle-end state
Inlet flow BHE_in_ 3 °C	Inlet flow BHE_in_ − 3 °C	Inlet flow: stop	Inlet flow BHE_in_ 3 °C
Return flow BHE_out_ 3 °C	Return flow BHE_out_ − 2 °C	Return flow: stop	Return flow BHE_out_ 3 °C
Vessel 3 °C	Vessel 3 °C	Vessel 3 °C	Vessel 3 °C
Steady state soil 3 °C	Freezing to steady state (> 38 h)	Thawing to steady state at 3 °C	Steady state soil 3 °C

## Results and discussion

### Influence of temperature changes

**Fig. 8:**
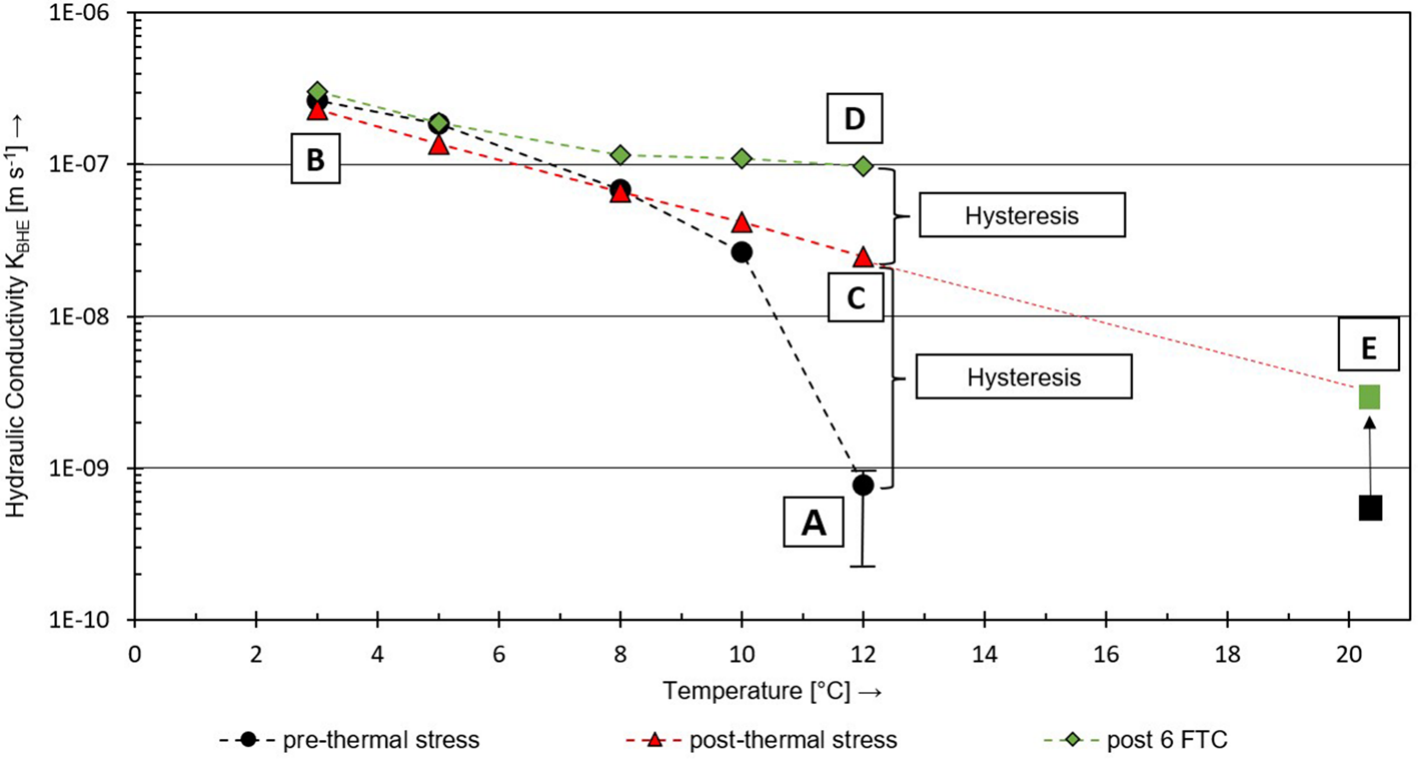
Mean values of hydraulic conductivity measurements of *K*_BHE_ at varying temperature steps with initial state (**A**), cooling process (black) to 3 °C (**B**), heating process (red) to 12 °C (**C**), heating process after FTC6 (green, **D**) and comparison values of a parallel experiment according to VDI 4640-2 (2019) with the same grout–pipe material combination at 20 °C, mean values of 3 specimen 600 readings each (**E**)

First of all, investigations were conducted to detect experimentally the influence of temperature changes on the hydraulic integrity of BHE under non-freezing conditions. Figure 8 illustrates the measurements of the hydraulic conductivity of the BHE (pipes and grout) *K*_BHE_ for the different temperature levels. It is obvious that the hydraulic system conductivity of the specimen in the pilot experiment is strongly dependent on the overall temperature level.

The initial hydraulic conductivity (A) at 12 °C was 7.7 × 10^–10^ m s^−1^, which is in the range of the expected magnitude and therefore in good agreement with the results for the initial state (black) from the norm experiments (E). When cooling the vessel (black marks) to 10 °C, an initial significant increase of *K*_BHE_ up to 2 × 10^−8^ m s^−1^ was observed. Ongoing cooling of the BHE pursued this trend and increased the system conductivity linear on the logarithmic scale up to (B) 2 × 10^–7^ m s^−1^ at 3 °C. The subsequent heating of the experiment in steps from 3 °C up to 12 °C confirmed this trend in reversed order. An exponential behavior of the hydraulic system conductivity depending on the overall temperature level was confirmed and resulted in *K*_BHE_ (C) ~ 2.5 × 10^–8^ m s^−1^ at 12 °C at least. However, a difference between the hydraulic system conductivity after the thermal loading and the initial hydraulic system conductivity of the BHE remained and resulted in an irreversible hysteresis of 1.5 magnitudes.

Following the temperature-level tests, the experiment was cooled down to 3 °C to carry out a sequence of FTC as presented in “Test routines” section. The hydraulic measurement with a stepwise increase of the temperature level was reproduced afterwards and proofed an FTC-induced increase of the BHE hydraulic conductivity up to a value of ~ 9.4 × 10^–8^ m s^−1^ at 12 °C (Fig. 8D).

Regarding the effect of the temperature changes on the hydraulic conductivity of a BHE system, consisting of pipes surrounded by grouting material, the presented results are in general good agreement with results reported by Allan (2000). It seems to be most feasible that the hydraulic conductivity increase is localized at the contact of hydrophilic grout to the hydrophobic PE-pipe and resulting from the stronger thermal shrinking of the pipe in opposition to the grouted body when decreasing the temperature.

Simultaneously, an experiment according to Anbergen et al. (2014) and VDI 4640-2 (2019) was carried out under ambient laboratory conditions (20 °C, Fig. 8E) using the same grout and pipe combination as installed in the pilot-scale experiment. The plotted values include the initial system permeability (black) and the system permeability after FTC_6_ (green). The results fit perfectly into the extrapolated course of the retrograde path of the pilot-scale experiment without FTC exposure. Consequently, the thermal conditions are expected to have a significant influence on the measured results for the Anbergen experiment as well.

**Fig. 9:**
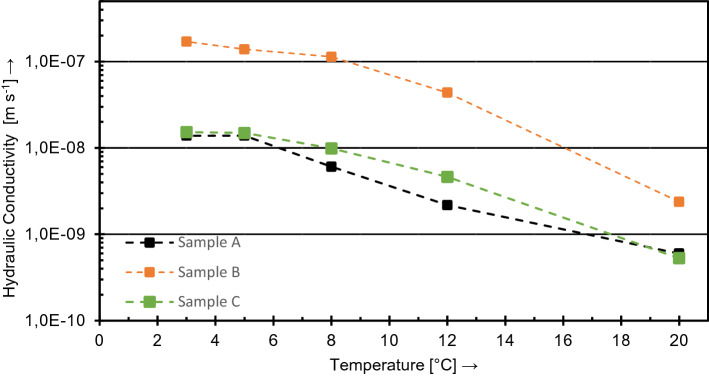
Mean value comparison of hydraulic conductivities of grout-pipe samples tested according to VDI 4640-2 (2019) (sample hardening for 56 days at 10 °C)

To double-check these results an additional experiment with the same pipe product but deviating grouting material was performed. For this purpose, three samples were cured for 56 days at a temperature of 10 °C according to VDI 4640-2 (2019). The general experimental conditions were similar to the ones of the pilot experiment. If changes were applied, it is mentioned herein. The overall influence of the temperature level on the hydraulic conductivity between grout and PE-HD pipe could also be observed in that particular additional experiment as shown in Fig. 9. Starting with the measurement under conventional laboratory conditions as specified by VDI 4640-2 (2019), the reduction of the temperature-level results in an increase of the hydraulic system conductivity by around 1.5 magnitudes, subsequently. However, the disproportional strong influence of the first temperature decrease, as shown in Fig. 8A, could not be reproduced. Although the samples were also cured at 10 °C, they were then installed into the test cells under laboratory conditions (20 °C) and thus already subjected to a temperature change which probably influenced the contact area between pipe and grout. The elevated initial level of hydraulic conductivity of specimen B can be explained by hairline cracks during sample preparation.

Considering the presented results, it is quite obvious that the annular space between PE-pipe and grout varies regularly as a result of differential thermal contraction and expansion of both materials. Thus, an increase of the system hydraulic conductivity by just regular operation of the BHE and without of any application of freeze–thaw stress seems to be very likely.

### Freeze–thaw cycles

To investigate the influence of freezing and thawing on the hydraulic integrity as well as the temperature distribution of a complete BHE system, consisting of heat exchanger pipes, grout and surrounding subsoil, the pilot experiment was subjected to six FTC. Although the individual test procedures are identical for the different FTC, the observed characteristics are not equally pronounced in each test. Thus, the results are presented and discussed exemplarily using the third FTC (FTC_3_), because in this FTC the main features are particularly prominent.

**Fig. 10:**
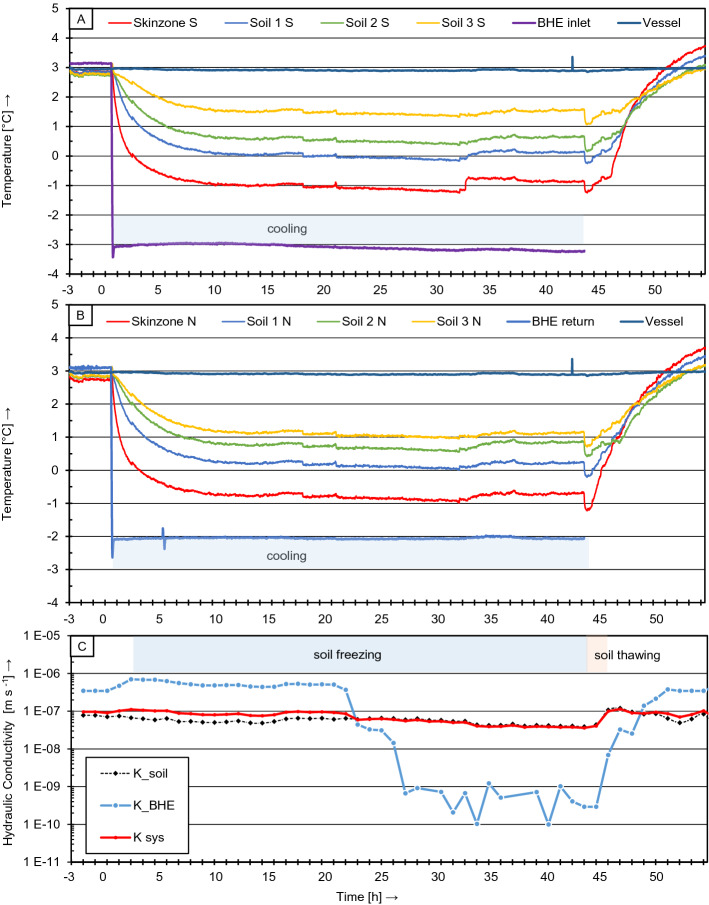
Measuring results of FTC_3_ out of FTC_6_ of the pilot-scale experiment. Upper plot: temperature measurements in southern orientation. Middle plot: temperature measurements in northern orientation Lower plot: hydraulic conductivity of BHE in blue (*K*_BHE_), soil conductivity in black (*K*_SOIL_) and the whole BHE system in red (*K*_SYS_). The results are displayed in dependence of the soil freezing phase and the thawing phase

Figure 10 illustrates the course of the temperature measurements as well as of the corresponding separately monitored hydraulic conductivities over the duration of FTC_3_.

FTC_3_ was started as any FTC from the pre-conditioning phase at a temperature of 3 °C, with a *K*_BHE_ of about 3.5 × 10^–7^ m s^−1^ and *K*_SOIL_ 8 × 10^–8^ m s^−1^, respectively. This in total resulted in a *K*_SYS_ of ~ 1 × 10^–7^ m s^−1^. Between 0 and 3 h the cooling of the specimen triggered thermal contraction of the components (mostly the pipe) and caused a minor increase of *K*_BHE_ to approximately 7 × 10^–7^ m s^−1^. After 5 h of cooling, the freezing of the specimen begun, which can be recognized by the temperature in sensor Skin zone *S* and *N* falling below 0 °C. After 20 h, ice formation initiates a reduction of the *K*_BHE_, which decreases by about three orders of magnitude in the following hours.

When the thawing process was initiated after 40 h, the *K*_BHE_ value began to rise again, approaching almost its initial state at the end of the FTC (after about 48 h). In contrast to *K*_BHE_, the soil hydraulic conductivity (*K*_SOIL_) only underwent minor changes over almost the entire pilot-scale experiment, with one exception: during the thawing of the sample column at around 42 h a moderate increase in *K*_SOIL_ was observed, very likely due to an opening of ice-clogged flow paths. The course of the hydraulic system conductivity (*K*_SYS_) indicates clearly that the changes to be observed depend solely on increase or decrease of *K*_SOIL_. This is no surprise, since in terms of the relevant water flow areas, the proportion of *A*_SOIL_ is significantly larger than that of *A*_BHE_.

The first temperature decrease at the beginning of the freezing operation ends after 8 to 10 h. From this point on, a plateau phase was observed for approx. 12 h. Afterwards (beginning after 20 h), the temperature further decreases simultaneously with the reduction in *K*_BHE_. A plausible explanation is that a lower heat transfer resistance between pipe and grout might exist, because the thermal conductivity of ice is four times higher than that of water. Furthermore, the convective heat transfer by water transport through the pores might be blocked by ice.

After a test duration of approx. 30 h, a sudden rise in temperature occurred in the skin zone, which was also detected in sensors 1 to 3 in an attenuated and delayed manner. This phenomenon is interpreted herein as a latent heat transfer effect. Due to the strong nature of the effect in the area of the skin zone, it is assumed that a certain amount of water was frozen in this area, which consequently suggests that there were distinct flow paths in this area. However, such a sudden temperature rise cannot be observed in the direction of the BHE return flow (Fig. 10B). Here the temperature curves during cooling show an overall flatter drop as well as smaller latent heat effects. This can be explained by the unequal temperature distribution due to the different temperatures in the inlet and return flow pipes.

As mentioned above, while freezing, a radial attenuation of the temperature rise induced by latent heat effects is obvious. In contrast, when the active cooling was stopped after 40 h, a sudden temperature drop was detected by all temperature sensors. This behavior may not be explained with latent heat effects. It is assumed that an unintended influence on the cold junction of the thermocouples occurred due to a decrease in the laboratory temperature of 3 K after switching off the active cooling. After this sudden temperature drop, a gradual temperature rise was established.

While on the return side (Fig. 10B) the temperature rise was more or less moderate (with the exception of Sensor Soil 2 N), on the inlet side (Fig. 10A), the temperature rise was initially attenuated for about 2 h before finally gathering momentum. This dampening effect was most likely caused by the reversal of the latent heat influence during the thawing process.

During further warming, the skin zone temperature shows a steeper rise than the temperatures in the soil. This is another indication for an increased permeability in the skin zone, which leads to an increased convective heat transfer with the water fed into the specimen at a temperature of 20 °C that acts as a thermal tracer. These results indicate that the increased permeability in the skin zone region is highly relevant to system considerations.

Comparing the values for the hydraulic conductivity of the soil and the BHE measured after various FTCs (Table 5), only minor variations are apparent. However, during the phases of active cooling and the dewing process itself, oscillations in the conductivity of the soil were detected. However, after these cyclic loads, the hydraulic conductivity usually approximated its initial state again.

**Table 5:** Hydraulic conductivities (m s^−1^) measured after each FTC at 3 °C as well as the measurements at 12 °C before and after the experiment (± calculated measurement uncertainty)

FTC	0	1	2	3	4	5	6
*K*_SOIL,12_	2.0 × 10^–8^ ± 2.6 × 10^–10^	–	–	–	–	–	–
*K*_BHE,12_	4.9 × 10^–10^ ± 3.9 × 10^–10^	–	–	–	–	–	9.4 × 10^–8^ ± 3.4 × 10^–9^
*K*_SOIL,3_	6.0 × 10^–8^ ± 1.5 × 10^–9^	6.8 × 10^–8^ ± 1.8 × 10^–9^	8.2 × 10^–8^ ± 2.1 × 10^–9^	4.5 × 10^–8^ ± 1.1 × 10^–9^	1.3 × 10^–7^ ± 2.7 × 10^–9^	5.4·10^–8^ ± 1.3 × 10^–9^	8.3 × 10^–8^ ± 2.1 × 10^–^
*K*_BHE,3_	1.8 × 10^–7^ ± 6.3 × 10^–9^	2.1 × 10^–7^ ± 7.5 × 10^–9^	2.2 × 10^–7^ ± 7.7 × 10^–9^	3.5 × 10^–7^ ± 8.1 × 10^–9^	2.5 × 10^–7^ ± 8.7 × 10^–9^	2.3 × 10^–7^ ± 8.1 × 10^–9^	2.0 × 10^–7^ ± 6.9 × 10^–9^

The increase in soil conductivity during cooling from 12 to 3 °C was caused by the loss of flexibility of the latex cover as the temperature had decreased. As a result, a circumferential flow developed on the outside of the sample, which could be proven afterwards with uranine markings.

### Visualization of impairments using uranine tracer

**Fig. 11:**
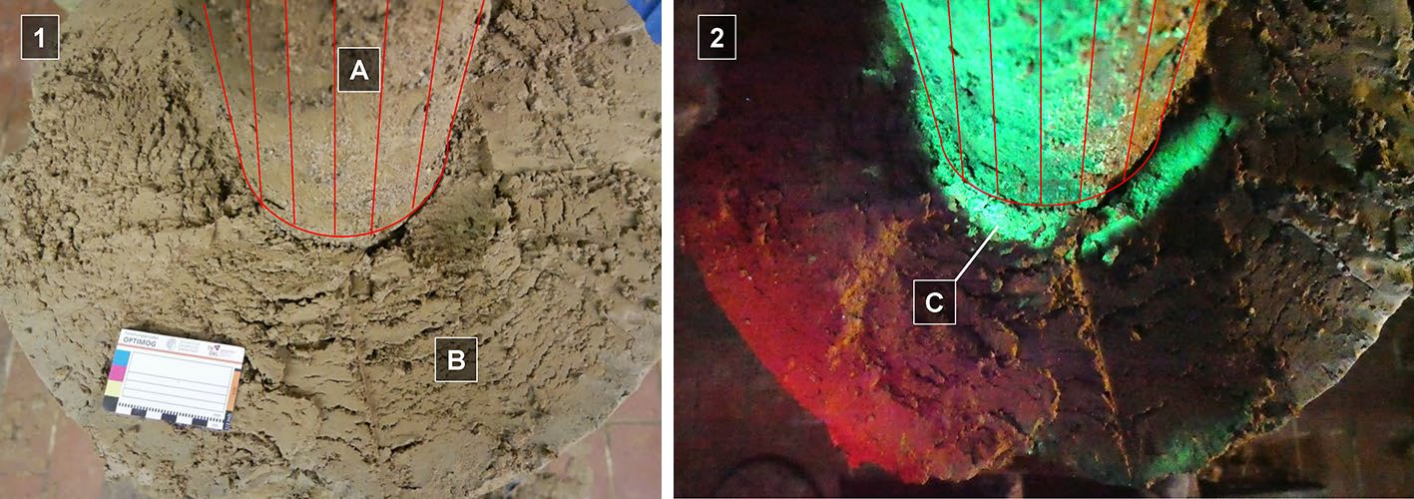
Plain view on the dismantled specimen of BHE body (**A**) and surrounding soil (**B**) at day light (1) and with black light (2) to visualize flow paths in the skin zone (**C**). The BHE body is drawn in red color in a schematic way to increase the visibility of the BHE grout

After finalizing the thermal experiments, a highly concentrated uranine solution was injected into the inlet of the sample flow to serve as a fluorescent marker for a qualitative assessment of the experiment. Under black light, preferred flow paths of the uranine tracer fluoresces can be visualized (Fig. 11).

Special attention should be paid to the skin zone, where uranine penetrated approx. 15–20 mm into the soil body, radially. This indicates that the soil in the vicinity of the BHE had a considerably increased hydraulic conductivity. The annular volume, which is affected by uranine impregnation, is congruent with the depth of the freezing front penetration as measured during the FTC tests. Consequently, freezing effects have an unfavorable influence on the hydraulic conductivity, not only on the BHE itself, but also on the surrounding natural subsurface.

**Fig. 12:**
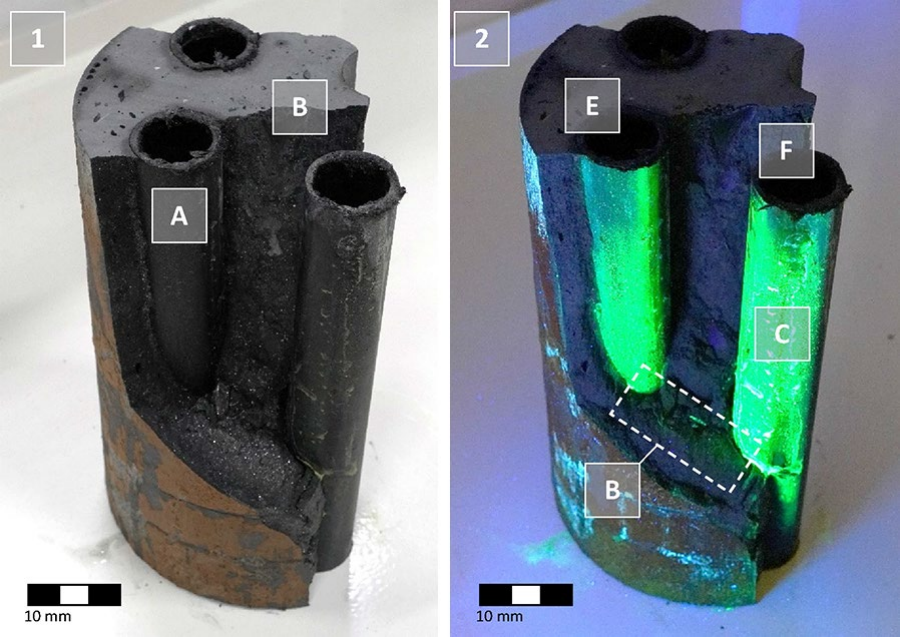
Plain view in the dismantled and washed BHE body of PE-HD pipes (**A**) and grout (**B**) under day light (1) and black light (2). Circumferential flow paths (**C**) at the PE-HD pipes and cracks (**D**) between return (**E**) and inlet flow (**F**) are clearly visible

Furthermore, the uranine method highlighted additional preferential flow paths in the grouted backfill of the BHE (Fig. 12): residues of uranine were detected on the outside of the pipes as well as on the contact surfaces at the grout.

If the observed increases in permeability in the skin zone gained a penetrative character, brittle material failure (cracks) caused by freeze–thaw effects were detected as a disintegration of the grout body. In particular, these effects occur between the supply and return pipes of the BHE, where the mechanical stresses are considered at a maximum due to the high temperature differences. Moreover, the effects of tensile stress were observed on the largest section of the specimen height, but the intensity was variable.

In conclusion, the experimental data, especially the hydraulic investigations, were confirmed by the qualitative visual observations using the uranine tracer.

## Transferability of the results

Like with any laboratory experiment, the one presented in this study was subject to simplifications and uncertainties, which impair the transferability of results.

The test procedure itself represented a major simplification of reality. It was characterized by defined boundary conditions, such as the starting temperature and the temperatures for heat extraction under cyclic loads. In reality, FTC are subject to natural fluctuations. However, reproducing natural fluctuations in such an experiment would cause severe noise, which would obscure important findings and render a reproduction of the results as impossible.

Another point of discussion is the sudden cooling at the beginning of the frost phase. In a real BHE operation, the cooling proceeds more moderately. Nevertheless, the maximum Δ*T* of 6 K in the presented experiment was rather gentle compared to the Anbergen experiment (Δ*T* = 30 K). Moreover, it is expected that the freezing process was still slowed down, enough to produce comparable freeze effects.

Furthermore, the active control of the vessel temperature with constant 3 °C did not necessarily correspond to the transient temperature behavior around a real BHE. This temperature is also based on previous modeling of a 100-m BHE and corresponds to the temperature distribution in a realistic reference scenario after 10 years of operation. The soil temperature was determined at peak loads within a radius of approx. 25 cm from the center of the BHE. This surely had an effect on the spread of the frost front because of an unnaturally large heat input over the vessel wall. Consequently, it is feasible that the propagation of the frost front was underestimated in this experiment. Nevertheless, an external tempering was necessary to ensure transferable conditions according to reality during the FTC and thawing from the outside to the inside. Moreover, assuming steady-state conditions and taking the soil thermal conductivity into account, the temperature gradient from the vessel wall to BHE induced a radial heat flow of approximately 40 to 50 W/m. This is very well within the range of a real BHE in peak load operation.

The hydraulic conditions for the test were determined according to DIN EN ISO 17892-11 (2019) as well as empirical values. However, it was challenging to adjust a viable pair of supply pressure and back pressures (i.e., the hydraulic gradient). It had to be, on the one hand, high enough to facilitate a quick saturation and consistent results in a reasonable time, and, on the other hand, low enough to avoid sample damage due to suffusion. The fact that in this experiment, in contrast to many real BHEs, there is no backfill pipe in the sample cross section, an underestimation of the hydraulic system permeability can be assumed.

Another limitation of the experiment relates to the pressure conditions. While a real BHE extends over several tens of meters and thus a wide range of pressure conditions, the experiment only represents a certain depth section. Of course, it would be of utmost interest to conduct multiple experiments with different boundary conditions representing different depth sections, however, the time-consuming preparation and performance of the experiment are defining operational and financial limits to the execution.

The test liquid, which perfused the specimen, was kept at laboratory temperature (20 °C) in the hydraulic cylinders. This in turn meant an unnaturally high heat input into the specimen, which resulted in an underestimation of the frost front propagation. However, the comparatively high temperatures of the test liquid rendered it to be a thermal tracer. In conjunction with the sensor distribution, it provided valuable information about internal water paths in the sample.

When the sample was removed, uranine tracer was visually detected on the lateral surface of the sample. This proved the previous assumption that there had been a circumferential flow on the outside of the sample. The hydraulic conductivity of the soil itself was determined in a laboratory experiment to 1 × 10^–8^ m s^−1^. In contrast, the initial measurement in the pilot plant at 12 °C was 2 × 10^–8^ m s^−1^. Moreover, the conductivity increased to 6 × 10^–8^ m s^−1^ at a temperature of 3 °C. This is attributed to the fact that the latex sleeve was too inflexible and had, especially at lower temperatures, a limited sealing function. Nevertheless, the spread of the uranine marker on the outside of the specimen was clearly subordinate in intensity compared to the marker in the skin zone. Thus, it can be expected that the results of the experiment are valid.

As mentioned before, one major drawback of such an elaborate experiment is its extremely time-consuming nature. One experiment requires at least half a year excluding the time needed for planning, manufacturing and construction of the experiment’s infrastructure. It requires dimensions and a cost like a field survey. Nevertheless, it is planned to repeat the experiment with further configurations of deviating heat exchanger pipe and grout materials.

## Conclusions and outlook

The paper presents a novel pilot-scale testing system to investigate the hydraulic integrity of a borehole heat exchanger under in situ conditions. For the first time, not only the borehole heat exchanger pipes and the backfill material, but also the skin zone and the surrounding unconsolidated rock are included in the consideration.

The experiments carried out within this study focused on the investigation of thermal effects on the hydraulic system permeability of a BHE installed in a low-permeability soil. Two different operational states were regarded.

In a first experimental phase, the behavior of the system in a regular operation with fluid temperatures above the freezing point of water was investigated. An important finding is that already this regular non-freezing operation of a BHE within temperatures between 0 and 12 °C can lead to a perceptible increase of the system’s vertical hydraulic conductivity. This is attributed to a relatively high thermal expansion factor of the PE-HD pipes compared to the cured grout body. A certain portion of this hydraulic conductivity gain is irreversible and has been experimentally identified as permeability hysteresis.

In a second experimental phase, an operation with cyclic loading and regular fluid temperature drops below − 3 °C inducing freezing and thawing processes in the vicinity of the BHE was experimentally reproduced. This operation led to further significant permeability increases, which are attributed to impairments caused by the formation of ice in the contact zone between pipe and grout, and the pores of the grout.

More importantly, zone-related permeability measurements showed that the FTC-induced permeability increase is not limited to the BHE body itself, but also extends into the soil body. Subsequent uranine marking of the flow paths revealed that this increase was basically concentrated in the skin zone.

When the permeability measurements of the BHE are conducted at temperatures just above the freezing point, the main flow still takes place on the thermal expansion-induced annular gap. Consequently, the FTC-induced impairment is only measurable at increasing temperatures, when the main flow gap has been closed again by the pipe expansion.

Comparing the results gained in the pilot-scale experiment to results of the standard test for hydraulic system integrity after FTC according to the VDI 4640-2 (2019) guideline leads to the following conclusions:

- The standard test according to the VDI 4640-2 (2019) is conducted with one single BHE pipe and thus represents a simplification of the real BHE geometry. Hence, it is not capable to reproduce the actual asymmetric temperature distribution in the grout body. Nevertheless, the standard test according to the VDI 4640-2 (2019) generally agrees well with the test results.
- An important disadvantage of the standard test is that the permeability measurements are usually carried out at laboratory temperature. Such a practice does not reflect the permeability increase caused by the thermal expansion of the materials as described above. Consequently, it underestimates the system’s hydraulic conductivity at in situ temperature conditions. Therefore, it is highly recommended to adapt the standard test procedure and prescribe more realistic temperature conditions.

The results reveal the disadvantages of combining a rigid backfill and a “flexible” BHE pipe material. The standard grouting materials for geothermal applications usually are a mixture of cement, sand and bentonite. They shall combine the highest possible compressive strength with a minimal hydraulic and enhanced thermal conductivity. In contradiction the backfill has to be resistant against temperature fluctuations and freezing–thawing behavior. Additionally, a long-term plasticity of the backfill would be desirable. Grouting products which combine the aforementioned properties with plasticity maintained over the long term are not presented yet. Such a development can be a possible future research task.

An additional scientific challenge may be the improvement of the system integrity by improving the grout–pipe interface. One track in this direction can be the reduction of the hydrophobicity of the BHE pipes. Another track can be the structural change of the pipe’s surface to extend the flow paths. A third research track could investigate the material-specific thermal expansion coefficients. Also, using aluminum or stainless-steel pipes are of a certain interest because of high thermal conductivities and less hydrophobic properties. However, this would put the corrosion issue on the future research agenda and also raise cost issues.

## Data Availability

The datasets gained and analyzed during the current study are available from the corresponding author on reasonable request.

## Abbreviations

BHE: Borehole heat exchanger
PE-HD: High-density polyethylene
FTC: Freeze–thaw cycle

## List of symbols

*A*: Cross-sectional area (m^2^)
*A*_BHE_: Area of the BHE fraction of the specimen (m^2^)
*A*_GAP_: Area of the gap between pipes and grout (m^2^)
*A*_grout_: Area of the grout fraction of the specimen (m^2^)
*A*_pipes_: Area of the BHE pipes (m^2^)
*A*_skin_: Area skin zone of the specimen, mixture of grout and soil (m^2^)
*A*_soil_: Area of the soil fraction of the specimen (m^2^)
*A*_sys_: Area of the whole system (m^2^)
*i*: Hydraulic gradient (–)
*K*: Hydraulic conductivity **(**m s^−^^1^)
*K*_BHE_: Hydraulic conductivity of the BHE fraction of the specimen (m s^−^^1^)
*K*_soil_: Hydraulic conductivity of the soil fraction of the specimen (m s^−^^1^)
*K*_sys_: Hydraulic conductivity of the whole system (m s^−^^1^)
*L*: Sample height (m)
*P*: Pressure (kPa)
*t*: Time (s)
*Q*: Water volume flow (m^3^ s^−^^1^)
*Q*_BHE_: Water volume flow in the BHE fraction of the specimen (m^3^ s^−^^1^)
*Q*_GAP_: Water volume flow in the gap between pipes and grout (m^3^ s^−^^1^)
*Q*_grout_: Water volume flow in the grout fraction of the specimen (m^3^ s^−^^1^)
*Q*_skin_: Water volume flow in the skin zone fraction of the specimen (m^3^ s^−^^1^)
*Q*_soil_: Water volume flow in the soil fraction of the specimen (m^3^ s^−^^1^)
*Q*_sys_: Water volume flow in of the specimen in total (m^3^ s^−^^1^)
*v*: Area related flow rate (m s^−^^1^)
*V*_BHE_: Water flow volume in the BHE fraction (m^3^)
*V*_soil_: Water flow volume in the soil fraction (m^3^)
*α*_T_: Temperature correction factor for water viscosity (–)
Δ*h*_w_: Water head difference (m)
*σ*1: Effective stress *Z* axis (Pa)
*σ*2: Effective stress *X* axis (Pa)
*σ*3: Effective stress *Y* axis (Pa)
